# Extravascular stent management for migration of left renal vein endovascular stent in nutcracker syndrome

**DOI:** 10.1186/s12894-015-0063-0

**Published:** 2015-07-24

**Authors:** Lu Tian, Shanwen Chen, Gaoyue Zhang, Hongkun Zhang, Wei Jin, Ming Li

**Affiliations:** Department of Vascular Surgery, the First Affiliated Hospital of Medical College, Zhejiang University, Hangzhou, 310003 China; Department of Urology, the First Affiliated Hospital of Medical College, Zhejiang University, No. 79 Qing Chun Road, HangZhou, 310003 China; Department of Urology, the Second Affiliated Hospital of Zhejiang Chinese Medical University, Hangzhou, 310005 China

**Keywords:** Nutcracker syndrome, Stent migration, Management

## Abstract

**Background:**

Nutcracker syndrome is an entity resulting from left renal vein compression by the aorta and the superior mesenteric artery, which leads to symptoms of hematuria or left flank pain. The alternative option of endovascular or extravascular stenting is very appealing because of the minimal invasive procedures. Stents in the renal vein can cause fibromuscular hyperplasia, proximal migration or embolization.

**Case presentation:**

A 30-year-old female was diagnosed with nutcracker syndrome for severe left flank pain. After failed conservative approach, she underwent endovascular stenting and subsequently developed recurrent symptom for stent migration one month postoperatively. She underwent successful extravascular stenting with complete symptom resolution.

**Conclusion:**

The extravascular stenting is an alternative option after migration of left renal vein endovascular stenting. The computed tomographic imaging was closely correlated to therapeutic interventions and stent migration.

## Background

Left renal vein (LRV) compression by the aorta and the superior mesenteric artery (SMA) leading to symptoms of hematuria or left flank pain has been classically described as nutcracker syndrome (NCS) [1, 2]. Minimal invasive management includes both endovascular stenting and extravascular stenting [1, 2]. We reported a teaching case with NCS who underwent endovascular stenting and subsequently developed recurrent symptom for stent migration one month postoperatively. She underwent successful extravascular stenting with complete symptom resolution.

## Case presentation

A 30-year-old female was presented with severe left flank pain for one year. Laboratory data was within normal limits. Her physical examination was unremarkable, with a body mass index of 19 Kg/m^2^.

**Fig. 1:**
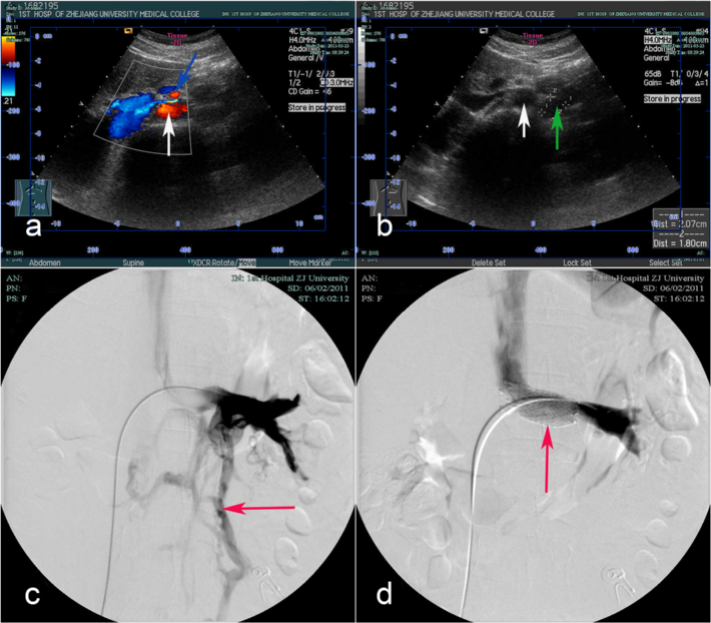
The images of the duplex ultrasound and the left renal venography. **a**, Right transverse image: Duplex ultrasound demonstrated the compressed left renal vein between the aorta (white arrow) and the superior mesenteric artery (blue arrow), and the left renal vein was pressed like a beak. **b**, Left transverse image: Duplex ultrasound demonstrated a narrowing of the left renal vein at the aortomesenteric portion and varices of left gonadal vein (green arrow) arising from the left renal vein on the left of aorta (white arrow). **c**, Before extravascular stenting, left renal venography demonstrated there was obstruction of left renal venous outflow and perihilar varices (red arrow). **d**, After endovascular stenting (red arrow), left renal venography showed unobstructed blood outflow and full stent expansion without obvious protrusion of the stent in the inferior vena cava

On April 8th, 2011, the computed tomographic angiography (CTA) and magnetic resonance angiography showed narrowing of the LRV in the aortomesenteric portion. On May 25th, 2011, a duplex ultrasound demonstrated the compressed LRV between the aorta and the SMA, varices of left gonadal vein arising from the LRV, and a peak velocity (PV) of 17 cm/s in the renal hilum and 106 cm/s in the aortomesenteric portion of the LRV (the PV ratio of 6.2) (Fig. 1a, b). On June 2th, 2011, left renal venography revealed obstruction of LRV outflow, perihilar varices, and an 8 mm Hg pressure gradient across the suspected narrowing in the LRV (Fig. 1c).

After failed conservative approach, the left renal venography was performed under local anesthesia to confirm and manage the narrowing of the LRV. A 10 mm × 40 mm SmartControl stent (Cordis, Johnson & Johnson, USA http://www.jnj.com) was deployed. The left renal venography showed unobstructed blood outflow, and full stent expansion without obvious protrusion of the stent in the inferior vena cava (Fig. 1d). The patient had nearly immediate resolution of her symptom and was discharged on postoperative day 5.

**Fig. 2:**
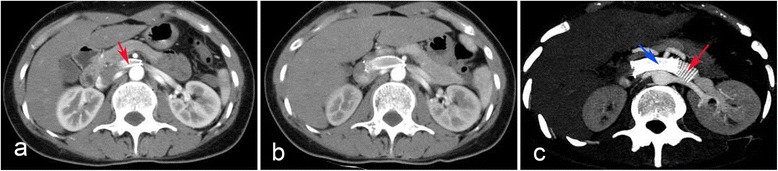
The images of the computed tomographic angiography (CTA). **a**, The second CTA evaluation was suggestive of an endovascular stent migration (red arrow) on the left of the superior mesenteric artery. **b**, The third CTA demonstrated further migration of the endovascular stent on the left of SMA. **c**, The follow-up CTA demonstrated the extravascular stent (red arrow) was patent and well positioned, and the endovascular stent (blue arrow) remained on the left of the superior mesenteric artery

**Fig. 3:**
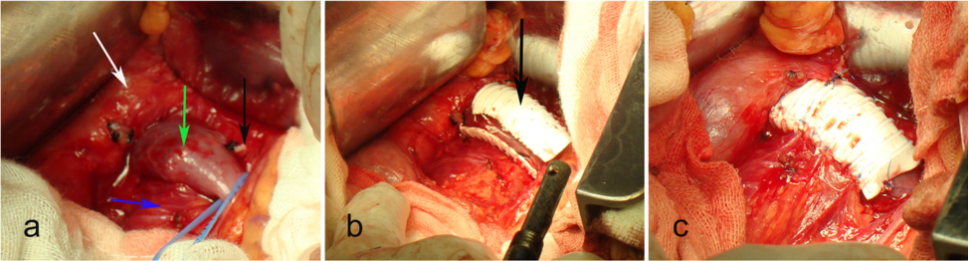
The images of the extravascular stent placement. **a**, The migrated endovascular stent was inside the left renal vein (green arrow), and the left adrenal central vein (black arrow) was ligated and transected. The aorta (blue arrow); the inferior vena cava (white arrow). **b**, Intraoperative photograph demonstrated the graft (black arrow) was wrapped around the renal vein. **c**, The graft was fixed together at each ring and sewn to the adventitia of the abdominal aorta

After one month of endovascular stenting, the patient began to experience recurrent left flank pain. On July 5th, 2011, the second CTA demonstrated an endovascular stent migration on the left of SMA (Fig. 2). On July 15th, 2011, the third CTA demonstrated further migration of the endovascular stent on the left of SMA (Fig. 2). Since there was a continuing migration of the stent on computed tomographic imaging within 10 days, the extravascular stent was proposed on July 26th, 2011. The endovascular stent was found migrated to the left of SMA and adhered to the vessel wall tightly, and the stent could not be moved. The varicose gonadal vein was seen arising from the LRV. Excessive fibrous tissue was found at the origin of the SMA, and excised for adequate decompression of the LRV (Fig. 3a). We estimated and cut the graft to an appropriate length to fit between the inferior vena cava and the gonadal vein or longer. After the left gonadal vein and adrenal central vein were ligated and transected (Fig. 3a), an externally reinforced polytetrafluoroethylene graft (REF F4008, Bard Peripheral Vascular, Inc. http://www.bardpv.com/) of 8 mm diameter was selected to form an extravascular stent around the LRV (Fig. 3b). The graft was wrapped around the LRV and fixed together at each ring (Fig. 3c). The graft was sewn to the adventitia of the abdominal aorta and the endovascular stent was sewn to the wall of the LRV to prevent from the further migration. The patient had nearly immediate resolution of her symptom and was discharged on postoperative day 7.

At 36 months’ follow-up, the patient was asymptomatic. The fourth, fifth and sixth CTA demonstrated the extravascular stent was patent and well positioned, and the endovascular stent remained to be on the left of the superior mesenteric artery at the first week, third month, and ninth month after extravascular stent placement respectively (Fig. 2).

## Discussion

Endovascular stenting has been used for seventeen years for the treatment of NCS due to its minimally invasive nature. A survey of the published English literature revealed 124 cases treated in this manner including our largest stenting experiences to date [2–9]. Although, the current literature suggests that stenting is a safe and effective procedure, stent migration notes in 7.3 % of all cases [2–5]. The reason of endovascular stent migration may be the effect of cardiac motion, early activity, mismatch between renal vein diameter or stent diameter, or inaccurate positioning of the stent within the lesion.

The clinical implications of migration are significant and can lead to thrombosis, vessel trauma, embolization, and its most disastrous consequence (rupture). It requires prompt and effective diagnosis and management to prevent potentially implications.

Sequence of image for diagnosis or follow-up has more or less been rationalized to duplex ultrasound, computerized tomography or magnetic resonance angiography, and finally left renal venography [2]. Duplex ultrasound is the easiest and the least expensive method. Zhelan Zheng et al. [10] pointed out standards for ultrasonic diagnosis of the disease as follows: (1)the low velocity of stenosis of the LRV at supine position accelerates remarkably, and the acceleration is more obvious after standing for 15 min,which is more than 100 cm/s; (2) the inner diameter ratio between renal hilum and stenosis of the LRV at supine position is more than 3, while it is more than 5 after standing for 15 min. When two index are coincident with the standards, NCS may be primary diagnosed. The CTA (including non-invasive 3-D) may be a useful tool in the diagnosis of the NCS and follow-up testing. CTA provided fine outlines that gave a precise depiction of both endovascular stent migration on the left of the SMA and a compression of the LRV between the aorta and the SMA. Furthermore, the stent migrating distance can be measured, and many distorting collateral veins were seen arising from the LRV in the CTA. The CTA imaging was closely correlated to therapeutic interventions and stent migration.

The typical treatment is percutaneous removal of the migrated stent. However, under certain circumstances, such as stent migration to the heart, special stent, or endothelialization of stent, percutaneous removal may be difficult or even impossible, thus surgery may be required. Hartung et al. described a LRV stent that migrated into the retro hepatic inferior vena cava; an attempt to retrieve it with a Goose Neck failed when the stent took a transversal orientation after 5 cm, and further attempts also failed [4]. A patient with a nitinol stent is difficult to manage percutaneously because of its inherent characteristics and probable endothelialization of the stent in 1 year, which makes the procedure more challenging [11]. In our previous case, one stent migrated into the right atrium and the patient required surgery after unsuccessful percutaneous removal [3]. In such cases, surgical removal is a safer and more feasible option. However, surgical removal is associated with high morbidity: Long period of renal congestion and additional anastomoses. Compared with surgical removal, extravascular stenting is a minimally invasive treatment modality.

Compared with vascular displacement, extravascular stenting for NCS is a minimally invasive treatment modality. Especially for children and adolescents, intravascular stenting should be cautiously recommended because the lumen of the LRV may become wider and the stents cannot match any longer during physical development. One may postulate that externally suturing stent could be a way to keep it in place; therefore, Barnes firstly reported extravascular stenting and externally suturing the stent performed by open surgery in 1988 [12]. Currently, sporadic cases of extravascular stenting for the NCS have been reported with excellent outcome at short-term follow up [13–17]. The stent has good conformability to adapt to the vessel wall and adhere to the vessel wall tightly [6]. In our opinion, the extravascular approach to treat endovascular stent migration is favored to avoid the potential complications.

Consideration must also be given to the original stent placement. If removal is not possible or failed, the original stent should be fixed to prevent repeated movements of the stent. Both the new and old stents should be sewn to the vessel wall to ensure that the extravascular and endovascular stents did not migrate, as shown in our case.

## Conclusions

The extravascular stenting is an alternative option after migration of left renal Vein endovascular stenting. The computed tomographic imaging was closely correlated to therapeutic interventions and stent migration.

## Consent

Written informed consent was obtained from the patient for publication of this manuscript and accompanying images. A copy of the written consent is available for review by the Editor-in-Chief of this journal.

## Abbreviations

LRV: Left renal vein
SMA: Superior mesenteric artery
NCS: Nutcracker syndrome
CTA: Computed tomographic angiography
PV: Peak velocity
